# Chloroquine and Rapamycin Augment Interleukin-37 Expression via the LC3, ERK, and AP-1 Axis in the Presence of Lipopolysaccharides

**DOI:** 10.1155/2020/6457879

**Published:** 2020-02-10

**Authors:** Xiaoyi Shi, Chunhui Lai, Lianyu Zhao, Mingying Zhang, Xi Liu, Shanqin Peng, Weizhong Guo, Qiuying Xu, Song Chen, Guang-xing Chen

**Affiliations:** ^1^Guangzhou University of Chinese Medicine, Guangzhou 510405, China; ^2^Division of Rheumatology and Clinical Immunology, The First Affiliated Hospital of Guangzhou University of Chinese Medicine, Guangzhou 510405, China; ^3^Science and Technology Innovation Center, Guangzhou University of Chinese Medicine, Guangzhou 510405, China

## Abstract

IL-37 is a cytokine that plays critical protective roles in many metabolic inflammatory diseases, and its therapeutic potential has been confirmed by exogenous IL-37 administration. However, its regulatory mechanisms remain unclear. U937 cells were treated with autophagy-modifying reagents (3-MA, chloroquine, and rapamycin) with or without LPS stimulation. Thereafter, IL-37 expression and autophagic markers (Beclin1, P62/SQSTM1, and LC3) were determined. For regulatory signal pathways, phosphorylated proteins of NF-*κ*B (p65 and I*κ*B*α*), AP-1 (c-Fos/c-Jun), and MAPK signal pathways (Erk1/2 and p38 MAPK) were quantified, and the agonists and antagonists of MAPK and NF-*κ*B pathways were also used. Healthy human peripheral blood mononuclear cells were treated similarly to confirm our results. Four rhesus monkeys were also administered chloroquine to evaluate IL-37 induction *in vivo* and its bioactivity on CD4 proliferation and activation. IL-37 was upregulated by rapamycin and chloroquine in both U937 cells and human PBMCs in the presence of LPS. IL-37 was preferentially induced in autophagic cells associated with LC3 conversion. AP-1 and p65 binding motifs could be deduced in the sequence of the IL-37 promoter. Inductive IL-37 expression was accompanied with increased phosphorylated Erk1/2 and AP-1 and could be completely abolished by an Erk1/2 inhibitor or augmented by Erk1/2 agonists. In monkeys, chloroquine increased IL-37 expression, which was inversely correlated with CD4 proliferation and phosphorylated STAT3. IL-37 levels were induced by rapamycin and chloroquine through the LC3, Erk1/2, and NF-*κ*B/AP-1 pathways. Functional IL-37 could also be induced *in vivo*.

## 1. Introduction

Interleukin 1 (IL-1) family member 7 (IL-1H4/IL-1F7b) was first identified in 2000 by Kumar et al. [1] and was recently renamed as IL-37 (isoform IL-37b) [2]. All IL-1 family members share a similar *β*-barrel structure and bind to Ig-like receptors [3], and most of them are proinflammatory cytokines including IL-1*α*, IL-1*β*, and IL-18 [4]. In contrast, IL-37 has been identified as a fundamental suppressor of innate immunity [5], which can either be secreted to act extracellularly or translocate to the nucleus and downregulate other proinflammatory cytokines such as IL-1*β*, tumor necrosis factor *α* (TNF-*α*), and IL-18 [5–7]. IL-37 can also act as an inhibitor of adaptive immunity via induction of tolerogenic dendritic cells [8]. Moreover, IL-37 reverses the metabolic cost of inflammation, increases oxidative respiration, and improves exercise tolerance [9]. IL-37 has been associated with diseases such as rheumatoid arthritis, atopic dermatitis [10], inflammatory bowel disease [11], and systemic lupus erythematosus [12]. These advances indicate the promising therapeutic potential of IL-37 in conditions such as autoimmune disorders [13], ischemia-reperfusion injury [14], transplant rejection [15], and allergic diseases [16].

Manipulating IL-37 expression *in vivo* is an attractive therapeutic strategy alternative to exogenous IL-37 administration, especially considering its functions after translocation to the nucleus [6, 7], which require further understanding about IL-37 regulation. IL-37 expression is endogenously kept at low levels in human cells and can be upregulated by various TLR agonists and proinflammatory cytokines including IL-1*β*, TNF-*α*, and IFN-*γ* [5]. TGF-*β* is the most effective stimulus for IL-37 induction whereas IL-4 and GM-CSF inhibit constitutive IL-37 expression [5]. As for the cell signaling levels, P38 MAPK and extracellular signal-regulated kinase (ERK) 1/2 pathways might be involved in IL-37 production [17, 18]. However, proinflammatory cytokine-induced IL-37 expression should be considered a protective response rather than a potential for therapeutic application.

Autophagy is an important homeostatic process responsible for degrading intracellular organelles and protein aggregates via a process involving the delivery of cytoplasmic cargo to the lysosome [19]. Indeed, autophagy-related gene polymorphisms have been associated with the pathogenesis of several autoimmune and inflammatory disorders [20–22]. Recently, autophagy has been shown to regulate inflammation during infection [23]. Activation of autophagy by inflammatory signals limits IL-1*β* production by targeting ubiquitinated inflammasomes for destruction [24]. The interactions between IL-37 and autophagy could be important, and there are several reports describing that IL-37 reduces the expression of mTOR and induces autophagy [25, 26]. Nevertheless, whether and how autophagy affects IL-37 expression remain unknown.

In this study, we examined IL-37 expression in LPS-stimulated U937 cells and healthy human PBMCs treated with two clinically approved autophagy-modifying drugs, chloroquine and rapamycin. We also examined IL-37 expression in chloroquine-administered rhesus monkeys. Overall, we investigated IL-37 induction as a novel therapeutic mechanism for inflammatory and autoimmune diseases along with the possible signaling pathways involved in regulating IL-37 expression by autophagy-modifying reagents.

## 2. Materials and Methods

### 2.1. Human Monocytic Cell Line

Human U937 cells were purchased from American Type Culture Collection (ATCC, Maryland, USA). Cells were maintained in RPMI 1640 medium (Gibco, Grand Island, USA) supplemented with 1% penicillin/streptomycin (Gibco, Grand Island, USA) and 10% fetal bovine serum (Gibco, Grand Island, USA) at 37°C in a humidified incubator with 5% CO_2_.

### 2.2. Human Peripheral Blood Mononuclear Cells (PBMCs)

After providing written informed consent, one healthy staff member donated 10 ml of EDTA anticoagulated blood for PBMC isolation by density centrifugation (Ficoll-Paque) and immediate culture. The ethics committee of the “First Affiliated Hospital of Guangzhou University of TCM” approved the protocol (No.[2015]22) as a preliminary study.

### 2.3. Treatment in Cell Cultures

U937 cells were seeded in culture plates at a density of 500,000 cells/ml and were transformed to attached macrophage-like cells in the presence of 200 nM phorbol 12-myristate 13-acetate (PMA, Sigma-Aldrich, St. Louis, MO, USA) for 48 h. Lipopolysaccharide (Escherichia coli, o55:B5) was purchased from Sigma-Aldrich (St. Louis, MO, USA) and used at 200 ng/ml for stimulation. In some settings, cells were treated with autophagy-modifying reagents including 400 nM rapamycin, 20 *μ*M chloroquine, or 1 mM 3-methyladenine (3-MA) simultaneously with LPS stimulation. Human PBMCs were seeded at one million cells/ml and were treated with autophagy-modifying reagents and/or LPS similarly. In some U937 cell cultures, agonists and antagonists of the MAPK signaling pathway (LM22B-10, anisomycin, SB 203580, U0126, and SP600125) and NF-*κ*B pathway inhibitor BAY 11-7082 were also used to investigate the roles of MAPK and NF-*κ*B pathways in IL-37 expression. All the above chemicals were purchased from MedChem Express (NJ, USA). Cells were harvested at different predesigned timepoints after treatment and were subjected to mRNA quantitation, flow cytometry analysis, and western blot analysis. Cell-free culture supernatants were also collected at 24 h after treatment and stored at -80°C until use.

### 2.4. Animal Experiment

Colony-bred rhesus macaques (Macaca mulatta) of Chinese origin were obtained from Guangzhou Mokeyking Biotechnology Co. Ltd. and were housed and maintained in accordance with the standards of the Association for the Assessment and Accreditation of Laboratory Animal Care (AAALAC) and the regulations of the *Guide for the Care and Use of Laboratory Animals* proposed by the National Institutes of Health. The Advanced BioScience Laboratories, Inc., Institutional Animal Care and Use Committee approved the protocol. All macaques were in good health, 2–4 years old, weighed 4–6 kg, and were seronegative for SIV, simian retrovirus, simian T cell leukemia virus type 1, and herpesvirus B. Four macaques were intragastrically administered 100 mg chloroquine diphosphate (C6628, Sigma) in 10 ml purified water, daily for seven consecutive days. At days 0, 3, and 7, EDTA-treated whole blood was collected to separate plasma and PBMCs. Plasma samples and PBMCs were stored at -80°C or in liquid nitrogen until use.

### 2.5. IL-37 Enzyme-Linked Immunosorbent Assay (ELISA)

IL-37 concentrations in the culture supernatants of both U937 cells and human PBMCs were measured using ELISA kits (Thermo Fisher, Vienna, Austria) according to the manufacturer's instructions. Plasma IL-37 levels in monkeys were also determined using this kit, and the results were apparently positive, though no validation data about its cross-activity are available.

### 2.6. RNA Isolation, Reverse Transcription, and Quantitative PCR

Treated U937 cells and PBMCs were subjected to RNA extraction using TRIzol reagent (Invitrogen, USA) and reverse transcription using the Super Script III First-Strand Synthesis System (Thermo Scientific, USA) according to the manufacturer's protocols. PCR was performed in triplicate using the TB Green qPCR master mix (Takara Biomedical Technology Co., Ltd, Dalian, China) on an ABI 7500 Real-Time PCR System (Thermo, USA). The cycling program was as follows: denaturation at 95°C for 15 s, 40 cycles of 95°C for 15 s, and extension at 60°C for 60s, followed by melt curve generation. The *ΔΔ*CT method was used to quantify relative mRNA levels as described in User Bulletin 2 (Applied Biosystems). The primer sequences used in this study are listed in Supplementary [Supplementary-material supplementary-material-1].

### 2.7. Flow Cytometry Analysis

After treatments with LPS and autophagy modifiers for 24 h and with Brefeldin A (eBioscience, San Diego, CA, USA) for the last 12 h, U937 cells and healthy human PBMCs were harvested and subjected to intracellular staining with primary antibodies including mouse anti-human IL-37 (clone 37D12, Invitrogen, San Diego, USA), anti-IL-1*β*, and isotype IgG (BioLegend, USA), using the Fixation/Permeabilization Working Buffer (eBioscience) according to the manufacturer's manual. The stained cells were analyzed on a FACSCAN II flow cytometer with FACSDiva software (BD Biosciences, San Diego, CA, USA).

After thawing, the monkey PBMCs were subjected to surface staining with anti-human CD38-FITC (AT-1, StemCell Technologies, Vancouver, BC, Canada), HLA-DR-PE-Cy7 (G46-6), CD4-FITC or CD4-PE (M-T477), CD3-APC (SP34-2), and CD8-PerCP (RPA-T8), as well as to intracellular staining with anti-human Ki-67-PE (B56) and Fixation/Permeabilization Buffer as described above. All antibodies were purchased from BD Biosciences (USA). We also tried to use anti-human IL-37 antibody (clone 37D12) for intracellular staining of monkey PBMCs but failed due to poor cross-reactivity (data not shown).

### 2.8. Cell Sorting for Autophagic U937 Cells

PMA-transformed U937 cells were treated with LPS and autophagy-modifying reagents for 2 h and were then harvested and stained with Cyto-ID green autophagy detection kit 2.0 (Enzo Life Sciences Inc., Farmingdale, NY, USA) according to the manufacturer's instructions. The prepared samples were maintained in culture medium containing 5% FBS on ice until sorting. Both the Cyto-ID-positive and Cyto-ID-negative U937 cells were centrifuged immediately after sorting, followed by RNA isolation using the TRIzol reagent and gene expression quantitation as described above. The purities of sorted Cyto-ID-positive cells were higher than 95%.

### 2.9. Confocal Microscopy Examination

U937 cells were seeded on confocal specialized slides and PMA-transformed for 2 days. The adherent U937 cells were then treated as described above for 2 h, fixed with Immunol Staining Fix Solution (Biyuntian Inc., Shanghai, China), and permeabilized with PBS containing 0.001% Triton X-100. After blocking with 10% goat serum (Sigma-Aldrich, USA), U937 cells were incubated with anti-LC3 antibody (1 : 200, CST, USA) followed by Alexa Fluor® 488-conjugated goat anti-rabbit IgG antibody (Thermo Fisher Scientific) or solely incubated with Alexa Fluor® 647-conjugated anti-P62/SQSTM1 (1 : 200, Abcam, USA). Thereafter, U937 cells were mounted with ProLong Gold Antifade Mountant containing DAPI (Thermo Fisher Scientific Inc., USA) and were observed with a LAS AF inverted laser scanning confocal microscope (Leica Microsystems, Heidelberg, Germany) using the appropriate filters.

### 2.10. Western Blot

After treatment, adherent U937 cells were washed twice with cold PBS and harvested with cell scrapers. Total protein was extracted from the cells using RIPA buffer (Beyotime Biotechnology Inc., Shanghai, China) containing 1% phenylmethyl sulfonyl fluoride (PMSF, Beyotime), 1% cocktail of protease inhibitors (Beyotime), and 1% cocktail of phosphatase inhibitors according to the manufacturer's manual. Protein concentrations were determined using the BCA protein quantitation kit (Beyotime). The aliquots of protein samples were separated on 8%–12% SDS-PAGE gels (Bio-Rad, USA) and transferred to polyvinylidene difluoride membranes (Merck Millipore, Darmstadt, Germany). The membranes were blocked with 5% skim milk in TBST buffer (20 mmol/l Tris-HCL, 137 mmol/l NaCl, and 0.1% Tween-20) or with 5% bovine serum albumin (BSA) (Biosharp, Hefei, China) in TBST for phosphorylated proteins. The membranes were then incubated overnight with diluted primary antibodies at 4°C. The primary antibodies used in this study included rabbit anti-human SQSTM1/p62 (#5114, 1 : 1000), Beclin1 (#3495, 1 : 1000), LC3B (#3868, 1 : 1000), *β*-actin (#4970, 1 : 1000), Phospho-I*κ*B*α* (Ser32, #2859, 1 : 1000), Phospho-p65 (Ser536, #3033S, 1 : 1000), Phospho-p38 MAPK (Thr180/Tyr182, #9215, 1 : 1000), Phospho-Erk1/2 (Thr202/Tyr204, #4370, 1 : 1000), Phospho-c-Fos (Ser32, #5348S, 1 : 1000), and Phospho-c-Jun (Ser73, #3270, 1 : 1000), all purchased from Cell Signaling Technology. After washing with TBST buffer, the membranes were incubated with the appropriate concentration of horseradish peroxidase- (HRP-) conjugated secondary antibody (Thermo Fisher Scientific, #31460, 1 : 5000) for 2 h at room temperature. The proteins were detected with an enhanced chemiluminescence reagent (Thermo Fisher Scientific, Rockford, IL, USA) on the ChemiScope 6000 imaging system (Clinx Science Instruments Co. Ltd., Shanghai, China). Protein expression was quantified using ImageJ software [27].

### 2.11. Statistical Analysis

Data are presented as mean ± standard deviation (SD). One-way analysis of variance (ANOVA) was performed to analyze the differences between experimental groups. Spearman rank analysis was performed to check the relationship between two variables. Statistical analysis was performed using SPSS 18.0 software (SPSS, Chicago, IL, USA), and differences were considered statistically significant at a threshold of *P* < 0.05.

## 3. Results

### 3.1. Rapamycin and Chloroquine Upregulate IL-37 mRNA in U937 Cells in the Presence of LPS

**Figure 1:**
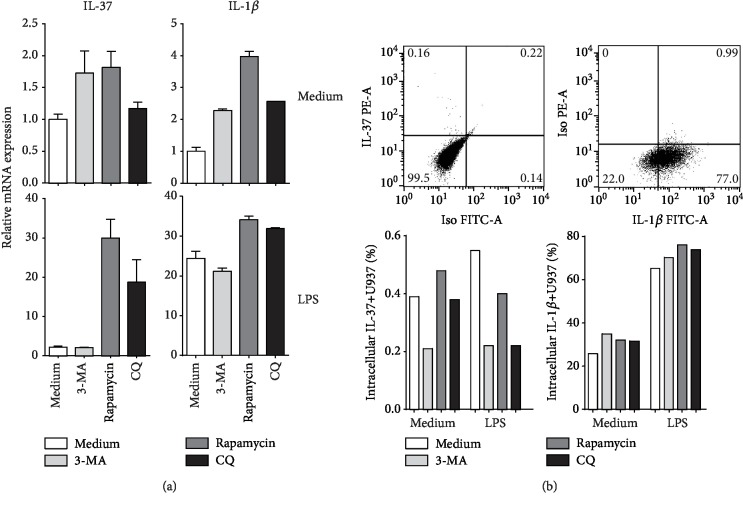
Effects of LPS and autophagy-modifying drugs on IL-37 and IL-1*β* expression in U937 cells. (a) Relative fold changes of IL-37 and IL-1*β* mRNA in U937 macrophages 2 h after treatments. Each sample was tested in triplicate, and the change folds were shown in Geomean ± S.E.M. (b) IL-37 and IL-1*β* intracellular staining in U937 macrophages at 24 h after treatments. Brefeldin A was used to block protein transport. The experiment was repeated twice and yielded similar results. The representative graphs of flow cytometry analysis are shown accompanied with IL37-positive and IL-1*β*-positive proportions in columns.

We first evaluated the effects of autophagy-modifying drugs on IL-37 expression in U937 macrophages. As shown in Figure 1(a), neither any autophagy-modifying drug nor LPS alone induced an obvious increase in IL-37 expression. Unexpectedly, upon LPS stimulation, both rapamycin and chloroquine treatments induced a remarkable increase (18.7-fold and 29.9-fold, respectively) in IL-37 mRNA expression. In contrast, the synergistic effects of LPS and autophagy-modifying reagents on IL-1*β* mRNA expression were much weaker. Moreover, using this culture system, we also observed the peak IL-37 expression at 2 h, which declined at 8 h after rapamycin treatment (data not shown). Therefore, we chose 2 h as the optimal timepoint in our further experiments. However, due to some unrevealed mechanisms, IL-37 production in U937 macrophages was extremely poor even with high IL-37 mRNA transcription and in the presence of Brefeldin A. In cultured U937 cells, only less than 1% treated U937 cells showed intracellular IL-37 staining at 24 h after treatment as determined by flow cytometry (Figure 1(b)), whereas the supernatant IL-37 levels were all undetectable by ELISA analysis (data not shown).

### 3.2. Chloroquine Augmented IL-37 Production in LPS-Stimulated Human PBMCs

**Figure 2:**
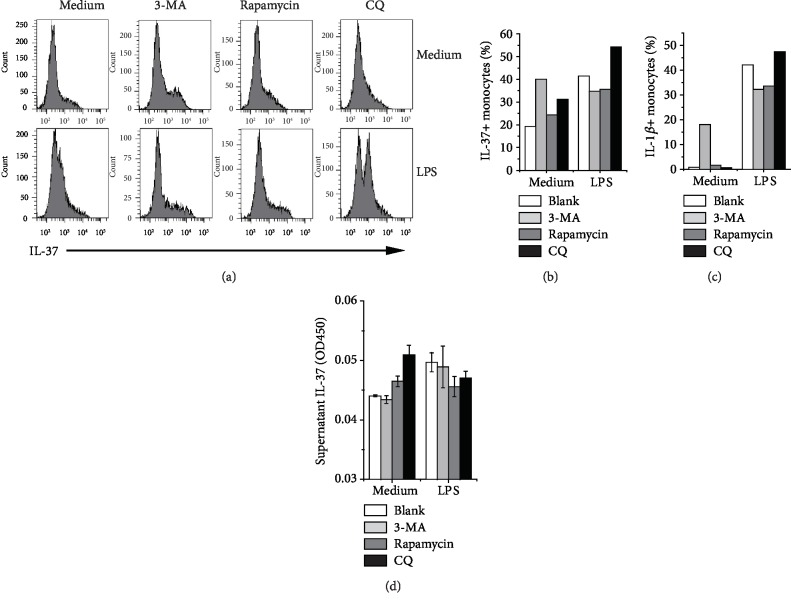
Effects of LPS and autophagy-modifying drugs on IL-37 and IL-1*β* expression in human PBMCs. (a) PBMCs from a healthy donor were isolated and treated with LPS and autophagy-modifying reagents for 24 h *in vitro*. Brefeldin A was also added to block protein transport for 12 h. Within the monocyte subgroups gated by their FS and SS characteristics, histograms of intracellular IL-37 staining detected by flow cytometry analysis are shown. (b) Columns indicate the proportions of intracellular IL-37-positive monocytes. (c) Columns indicate the proportions of intracellular IL-1*β*-positive monocytes. (d) Supernatant IL-37 levels determined by ELISA, and each sample was assayed in triplicate (Mean ± S.D.).

Due to poor IL-37 protein production in U937 cells, we isolated and cultured human PBMCs from a healthy donor to confirm our results. The IL-37-positive cells were predominantly monocytes as defined by their FS/SS characteristics. IL-37-expressing lymphocytes were very rare even after treatment with LPS and autophagy-modifying reagents (data not shown). As shown in Figures 2(a) and 2(b), LPS stimulation in combination with chloroquine treatment induced a remarkable increase in the proportion of IL-37-positive cells. Rapamycin showed weaker synergistic effects with LPS on IL-37 production compared to chloroquine, which might be explained by the protective effect of chloroquine on IL-37b mRNA, especially considering the presence of a coding region instability element in IL-37b mRNA [28]. Supernatant IL-37 concentrations were also quantified by ELISA. Although the OD450 values remained below the sensitivity threshold (31.3 pg/ml), they were apparently higher than those in the blank control. In our results, both chloroquine and rapamycin induced a slight increase in resting PBMCs, but not in LPS-stimulated PBMCs (Figure 2(d)). These results suggest that IL-37 processing and secretion might be differentially affected by chloroquine or rapamycin in the presence of LPS stimulation.

### 3.3. Chloroquine Increased IL-37 and Suppressed CD4 Proliferation *In Vivo*

**Figure 3:**
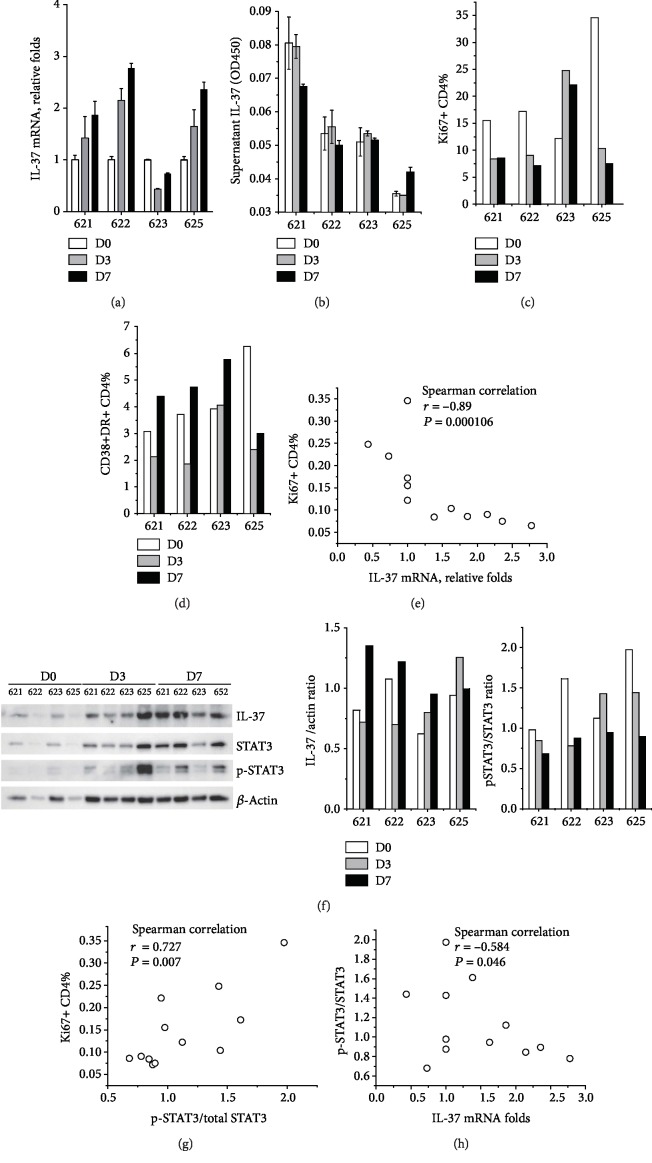
Effect of chloroquine administration on IL-37 expression, CD4 proliferation, and activation in rhesus monkeys. (a) Relative fold expression of IL-37 mRNA at 0, 3, and 7 days post chloroquine treatment in four rhesus monkeys. Each sample was assayed in triplicate and the change folds were shown in Geomean ± S.E.M. (b) Plasma IL-37 levels of monkeys were quantified by ELISA in triplicate (Mean ± S.D.). (c) Proportions of proliferating CD4+ T cells (Ki-67+CD4+CD3+) in peripheral blood at 0, 3, and 7 days post chloroquine treatment in monkeys. (d) Proportions of activated CD4+ T cells (CD38+HLA-DR+CD4+CD3+) in peripheral blood. (e) Spearman's correlation analysis between IL-37 mRNA and proportion of Ki-67+CD4. (f) Contents of IL-37, STAT3, and phosphorylated STAT3 in PBMCs of monkeys before and 3 and 7 days post chloroquine treatment. The experiment was repeated twice and yielded similar results. The representative western blot images are shown, and the ratio of IL-37 to housekeeping gene (actin) and the ratio of phosphorylated STAT3/STAT3 are shown in columns. Spearman's correlation analyses between (g) Ki-67+CD4 proportion or (h) IL-37 mRNA and phosphorylated STAT3 were also conducted and shown in graph.

As shown above, either chloroquine or rapamycin slightly decreased the supernatant IL-37 levels in cultured PBMCs though they increased the IL-37 expression in monocytes, whether IL-37 expression could be induced *in vivo* and be functional remained uncertain. As chloroquine induced higher IL-37 protein in monocytes compared to rapamycin, we evaluated the effect of chloroquine on IL-37 expression and CD4 proliferation and activation in rhesus macaques. As shown in Figure 3(a), chloroquine treatment at a daily dose of 100 mg caused a stable and gradual increase of IL-37 mRNA expression in the PBMCs of three out of four monkeys. Regretfully, due to poor cross-reactivity, we could not detect intracellular IL-37 staining in monkeys with the antibody clone 37D12 (data not shown). As no commercial monkey IL-37 ELISA kit was available, our ELISA kit (Thermo Fisher) was also used to determine monkey plasma IL-37 levels although its cross-reactivity had not been fully validated; ELISA showed weak but apparently positive results compared to the blank control. With this kit, no remarkable inductive IL-37 secretion could be observed even with increased IL-37 mRNA levels (Figure 3(b)). Nevertheless, the three monkeys with IL-37 mRNA upregulation showed an obvious decrease in proliferating (Ki-67+) CD4+ T cells (Figure 3(c)). Similarly, on day 3, the proportion of activated CD4 (CD38+HLA-DR+ %) was also remarkably decreased in the three monkeys accompanied with IL-37 mRNA upregulation but not in the exceptional monkey 623 whose IL-37 mRNA decreased with increased Ki67+ CD4+ T cells. However, activated CD4 in all monkeys unexpectedly rebounded on day 7 even though the IL-37 mRNA continued increasing at that timepoint (Figure 3(d)). A significant inverse correlation was observed between IL-37 mRNA and the proportion of proliferating CD4 with Spearman correlation analysis (*P* < 0.001) (Figure 3(e)). This result implied a profound inhibitory effect of IL-37 on CD4 proliferation. We also quantified the IL-37 contents in monkey PBMCs by western blot analysis. Typically, chloroquine treatment increased IL-37 protein levels in all four monkeys whereas the correlation between IL-37 protein and CD4 proliferation was not significant.

As IL-37b translocates to the nucleus, binds Smad3, and inhibits the phosphorylation of STATs 1-4 [5] and as tyrosine phosphorylated signal transducer and activator of transcription 3 (STAT3) is critical in T cell proliferation [29, 30], we further tried to explore the functional link of phosphorylated STAT3 between IL-37 and CD4 proliferation. Using western blot analysis, we showed that chloroquine treatment induced a gradual decrease in the ratio of phosphor-STAT3/STAT3, which was correlated with the proportion of Ki67-positive CD4 (*P* < 0.01) and inversely correlated to the relative IL-37 mRNA expression (*P* < 0.05).

### 3.4. Inductive IL-37 Expression Correlated with LC3 Conversion but Not P62

**Figure 4:**
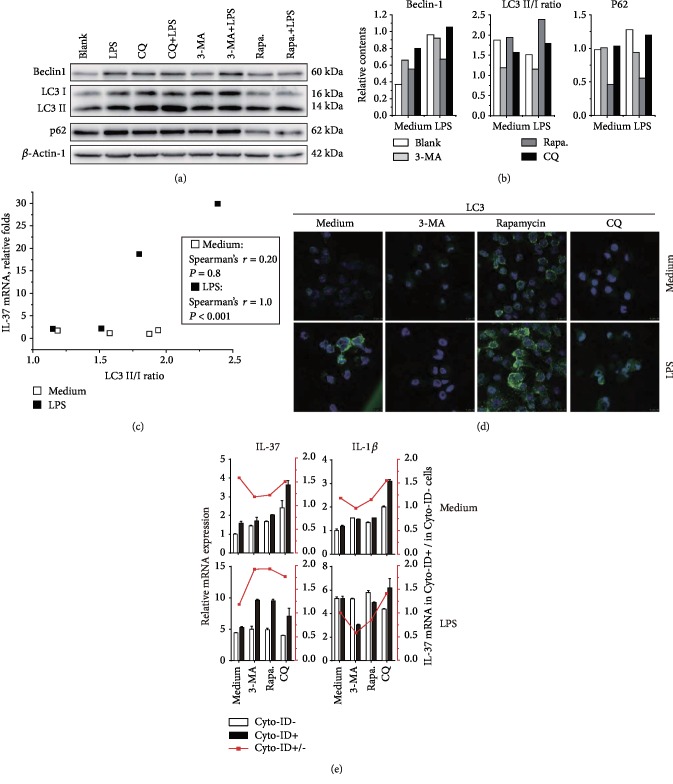
IL-37 expression induced by LPS and autophagy-modifying reagents correlates with LC3 conversion. (a) The representative western blot images and (b) columns of relative densitometric data showing the content of Beclin1, p62, and LC3 in treated U937 cells. The experiment was repeated twice independently and yielded similar results. (c) Spearman's analysis revealed a correlation between IL-37 mRNA and the LC3 II/I ratio only in the presence of LPS stimulation. (d) The representative images of LC3 immunofluorescence staining observed with a confocal microscope. (e) Comparison of IL-37 expression and IL-1*β* expression between Cyto-ID-positive cells and Cyto-ID-negative cells isolated by flow cytometry sorting (quantitation was repeated three times and presented as Mean ± S.D.). The ratio of IL-37 and IL-1*β* expression in Cyto-ID-positive cells to negative cells was also computed and plotted in the right.

As IL-37 mRNA expression was upregulated by both rapamycin and chloroquine, an inducer and an inhibitor of autophagy, respectively, it is reasonable to deduce that the accumulated intermediates of autophagy flux mediated the inductive transcription of IL-37. Therefore, using western blot analysis, we determined the contents of Beclin-1, p62, and LC3 and tried to identify the most relevant autophagic marker to IL-37 transcription. Among the three proteins, only LC3 conversion (LC3-II/I ratio) changed in a pattern similar to IL-37 mRNA after treatments with both autophagy-modifying reagents and LPS (Figures 4(a) and 4(b)). We further performed Spearman's correlation analysis and identified a significant correlation between the LC3-II/I ratio and the level of IL-37 mRNA in the presence of LPS stimulation, whereas the correlation did not exist without LPS (Figure 4(c)). On the contrary, the correlation between IL-37 mRNA level and P62 or Beclin-1 content was not significant. Consistent with the alteration of IL-37 mRNA levels, the fluorescence intensity of LC3 (Figure 4(d)) was also augmented after LPS stimulation and further strengthened by CQ or rapamycin treatment as determined by confocal microscopy analysis. In contrast, there was no obvious increase in P62 fluorescence intensity after LPS plus CQ or rapamycin treatments compared to LPS treatment alone (Supplementary Fig. [Supplementary-material supplementary-material-1]). Therefore, we speculate that LC3 conversion correlates with IL-37 expression, in which LPS stimulation plays a critical role.

Nevertheless, whether IL-37 transcription was exclusively induced in autophagic cells remained uncertain even though IL-37 mRNA levels in LPS-stimulated U937 correlate with LC3 conversion. Therefore, the autophagic cells were labeled with Cyto-ID staining, purified by flow cytometry sorting, and directly subjected to IL-37 mRNA quantitation. Cyto-ID selectively labels accumulated autophagic vacuoles with minimal staining of lysosomes. As shown in Supplementary Fig. [Supplementary-material supplementary-material-1], both the proportion of positive cells and the mean fluorescent intensity (MFI) of Cyto-ID were decreased in LPS-treated cells, implying the irrelevant role of autophagy in the upregulation of IL-37 mRNA induced by LPS alone. However, after treatment with LPS and autophagy-modifying reagents (3-MA, CQ, or rapamycin), IL-37 transcription was exclusively increased in Cyto-ID-positive cells but not in Cyto-ID-negative cells, which increased the ratio of IL-37 mRNA levels in Cyto-ID (+) cells versus that in Cyto-ID (-) cells (Figure 4(e)). Interestingly, the boosting effect was LPS stimulation-dependent as none of the autophagy-modifying reagents alone could increase IL-37 mRNA in autophagic cells (Cyto-ID +) without LPS stimulation, and this was specific for IL-37 transcription but not for IL-1*β* transcription (Figure 4(e)).

### 3.5. Rapamycin and Chloroquine Augment IL-37 Expression through the NF-*κ*B p65-Erk1/2-AP-1 Pathway

We further tried to explain the synergistic effect of LPS and autophagy-modifying reagents on the regulation of IL-37 transcription. As described elsewhere, LPS stimulation of human monocytes activates several intracellular signaling pathways that include the NF-*κ*B, MAPK, and AP-1 (c-Fos/c-Jun) pathways, which coordinate the induction of many genes encoding inflammatory mediators [31]. Considering that LPS stimulation is indispensable for inductive IL-37 transcription, we deduced that activation of NF-*κ*B, MAPK, and AP-1 pathways might be enhanced by autophagy-modifying reagents and in turn coordinate IL-37 induction.

In order to confirm this hypothesis, sequences of IL-37 promoter (2000 bp sequence immediately upstream of the IL-37 gene transcription start site) were searched for putative binding sites of NF-*κ*B and AP-1 transcription factors using JASPAR database (http://jaspar.cgb.ki.se), an open-access database of matrix-based nucleotide transcription factor binding profiles [32], and all NF-*κ*B and AP-1 matrices were included. With the “relative profile score threshold” as 90%, three putative AP-1 binding sites were found within the 221-263 bp region of the IL-37 promoter in the positive strand (Supplementary [Supplementary-material supplementary-material-1]). The corresponding matrix profiles, MA0099.2 and MA0476.1, are shown in Supplementary Fig. [Supplementary-material supplementary-material-1]. Further, plenty of NF-*κ*B binding sites as well as other AP-1 binding sites were found if we decreased the threshold to 80% (data not shown).

**Figure 5:**
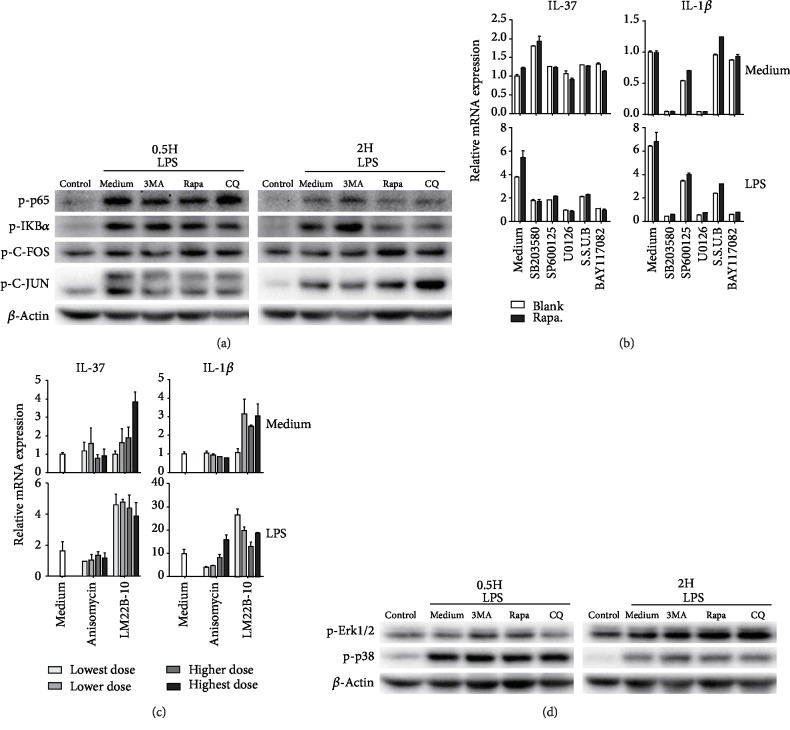
Cell signaling pathways contributing to the inductive transcription of IL-37. (a) The effect of LPS and autophagy-modifying reagents on the contents of phosphorylated NF-*κ*B p65/I*κ*B*α* and phosphorylated AP-1 (c-Fos/c-Jun) proteins. Two independent experiments were repeated and yielded similar results. The representative western blot images were shown. (b) The effect of MAPK inhibitors and NF-*κ*B inhibitor on the expression of IL-37 and IL-1*β* induced by LPS and rapamycin treatment in U937 cells. Each sample was assayed in triplicate, and the results were shown in Geomean ± S.E.M. Inhibitors used in this study included SB 203580 (100 *μ*M), U0126 (200 *μ*M), SP600125 (20 *μ*M), and BAY 11-7082 (10 *μ*M). S.S.U.B: combination of the four inhibitors. (c) The effect of MAPK agonists on IL-37, IL-1*β*, and IL-18 expression in untreated U937 macrophages was also investigated. The agonists were LM22B-10 at 125, 250, 500, and 1000 nM and anisomycin at 1.25, 2.5, 5, and 10 *μ*M. Each sample was assayed in triplicate (Geomean ± S.E.M.). (d) The effect of LPS and autophagy-modifying reagents on the contents of phosphorylated Erk1/2 and phosphorylated p38 MAPK. Two independent experiments were repeated and yielded similar results. The representative western blot images were shown.

Thereafter, we determined the contents of phosphorylated NF-*κ*B (p-p65 and p-I*κ*B*α*) and AP-1 (p-c-Fos and p-c-Jun) at 0.5 h and 2 h post treatments by western blot analysis. As shown in Figure 5(a), LPS stimulation induced a profound increase in both p-I*κ*B*α*/p-p65 and p-c-Jun and a slight increase in p-c-Fos at both 0.5 h and 2 h. In contrast, rapamycin treatment remarkably increased the p-c-Fos contents in LPS-stimulated cells at both 0.5 h and 2 h and increased the p-c-Jun content slightly at 2 h. In contrast, CQ most prominently increased p-c-Jun at 2 h and caused a mild increase in p-c-Fos. Treatment with 3-MA caused an apparent decrease in p-c-Jun content. Only CQ, but not rapamycin, induced a slight increase in p-p65 at 0.5 h, and both of them exerted no effect on p-I*κ*B*α*. These results suggest that differential activation of c-Fos and c-Jun could explain the effect of rapamycin and CQ to fuel IL-37 transcription at least partially. Activation of NF-*κ*B p65 only exerted weak effects, if at all, on inductive IL-37 transcription. The possibility that other transcription factors not discussed in this research also contributed to the fueling effect could not be excluded.

Considering the critical role of mitogen-activated protein kinases (MAPKs) in the regulation of AP-1 activity, we tried to evaluate the contributions of MAPK and NF-*κ*B pathways in IL-37 induction boosted by autophagy-modifying reagents. For this purpose, inhibitors targeting different three MAPK pathways (SB 203580 for p38, SP600125 for JNK, and U0126 for Erk1/2, respectively) and NF-*κ*B I*κ*B*α* (BAY 11-7082) were used. As shown in Figure 5(b), these inhibitors exerted no influence on IL-37 expression in resting U937 macrophages, indicating that the constitutive IL-37 expression is independent of the activation of MAPK or NF-*κ*B pathways. However, in LPS-stimulated U937 cells, induced IL-37 upregulation was diminished by all inhibitors and was nearly abolished by the Erk1/2 inhibitor, U0126. Moreover, the boosting effect of rapamycin on IL-37 transcription was also abolished. As combined treatment with SB 203580, SP600125, and BAY 11-7082 could not further decrease the IL-37 mRNA compared to treatment with U0126 alone, we deduced that the activation of Erk1/2 plays a central role in inductive IL-37 transcription. We also noticed that these antagonists could regulate autophagy due to the complicate reciprocal regulation between MAPK pathway and autophagy. Thus, partial suppression of IL-37 by SB 203580 and SP600125 might be explained by their inhibitory effect on the autophagy process.

Then, we used agonists of the JNK pathway (anisomycin) and Erk pathway (LM22B-10) to confirm our results. As shown in Figure 5(c), LM22B-10 (Erk activator) induced a significant increase in IL-37 transcription in both resting and LPS-stimulated U937 cells, whereas anisomycin exerted no effect. Using western blot analysis, the critical role of Erk1/2 activation in IL-37 induction was also supported by the increased content of phosphorylated Erk1/2 in LPS-stimulated U937 cells treated with either rapamycin or CQ (Figure 5(d)). The contribution of p38 MAPK activation to IL-37 induction might be excluded based on its poor response to rapamycin or CQ treatment. However, we noticed that 3-MA treatment also induced an increase in p-Erk1/2 without inductive IL-37 transcription. These results implied that Erk1/2 activation could be an indispensable factor but not a determinate factor for IL-37 induction.

For comparison, the effects of these agonists and antagonists on IL-1*β* expression were also examined in our study. As shown in Figures 5(b) and 5(c), SB-203580 and U0126 induced a profound decrease in IL-1*β* expression in both resting and LPS-stimulated U937 cells, indicating that both p38 MAPK and Erk1/2 critically contributed to both constitutive and LPS-induced IL-1*β* expression. Similarly, only LM22B-10 induced an obvious increase in IL-1*β* expression (Figures 5(b) and 5(c)).

## 4. Discussion

Macrophages play a critical role in the pathogenesis of inflammation and are the predominant source of IL-37 [33]. Consistently, using *in vitro* stimulated human PBMCs, we observed that intracellular IL-37 staining was dominantly enriched in monocytes. Moreover, we showed that up to 10-fold increase in IL-37 mRNA expression could be induced in PMA-stimulated U937 cells, indicating U937 cells as a suitable model for research on the transcriptional regulation of IL-37 although U937 cells produce poor levels of IL-37 protein. With this system, we observed that both chloroquine and rapamycin increased IL-37 transcription profoundly in the presence of LPS stimulation and identified a correlation between inductive IL-37 mRNA levels and LC3 II/I ratios. It is well-known that the crosstalk between autophagy and inflammatory signaling pathways balances defense and homeostasis [34]. Besides degradation of inflammation-related proteins, accumulation of autophagy intermediates such as P62/SQSTM1 [35–37] could serve as a scaffold to initiate multiple cell signaling pathways like NF-*κ*B [38, 39] and p38 signaling [40], and LC3 accumulation acts as a cellular scaffold to increase ERK phosphorylation [41]. ERK activation is known to upregulate IL-37 expression [18]. Consistently, using both an agonist and antagonist of the Erk1/2 pathway, we showed the critical role of Erk1/2 in IL-37 induction and observed an increase in the content of phosphorylated Erk1/2 after rapamycin and chloroquine treatments. In brief, our results emphasized a central role of LC3-II/Erk1/2 axis-mediated induction of IL-37 transcription by autophagy-modifying reagents.

Using JASPAR analysis, we found a number of putative AP-1 (c-Fos/c-Jun) and NF-*κ*B p65 binding sites clustered in the promoter sequences of IL-37. Moreover, we observed that LPS treatment caused a rapid and vigorous increase in the contents of phosphorylated p65 and that both chloroquine and rapamycin increased the contents of phosphorylated AP-1 (c-Fos/c-Jun). It is reported that c-Fos and c-Jun interact with NF-*κ*B p65 to form a complex with enhanced DNA binding and biological function via both the *κ*B and AP-1 response elements [42, 43]. This could be an explanation for the synergistic effect of LPS and chloroquine/rapamycin on IL-37 induction. Further, stimulation with LPS enhances the stability of the IL-37b transcript, as there is an instability element in the IL-37 ORF [28]. However, in our results, U0126, an inhibitor of Erk1/2, almost abolished the inductive IL-37 transcription suggesting that an increase in transcription rather than mRNA stability is more important for LPS-induced IL-37 mRNA upregulation.

Chloroquine has been frequently used in the treatment of inflammatory diseases including rheumatoid arthritis and systemic lupus erythematosus [44–47]. Rapamycin is also an immunosuppressive agent downregulating the expression of chemokines and has been widely applied for the treatment of rejection in organ transplantation, sarcomas, and in hematologic and solid malignancies [48, 49]. We speculate that IL-37 induction might be an anti-inflammatory mechanism shared by these two clinical immune-suppressive drugs. Therefore, we showed that in healthy rhesus monkeys, chloroquine treatment could gradually increase IL-37 mRNA and decrease CD4 proliferation and activation *in vivo.* This result provided direct evidence that functional IL-37 expression could be induced *in vivo*. Due to the ethical risks, we treated monkeys with chloroquine alone without LPS injection. However, we still observed a remarkable increase in IL-37 mRNA, which might be explained by the existence of low levels of LPS in the blood of healthy monkeys. Consistently, existence of LPS in the blood of healthy donors (approximately 0.128 ± 0.215 EU/ml) has been described previously [50, 51].

How IL-37 induced by chloroquine exerts its functions *in vivo* has not been fully elucidated. IL-37 is the only cytokine among proteins that bind Smad3 [52] and forms a functional IL-37- Smad3 complex, as Smad3 mostly antagonizes STAT1 and STAT3 [53]. Consistently, we observed an obvious decrease in phosphorylated STAT3 after chloroquine treatment in monkey PBMCs and identified a correlation between the ratio of phosphorylated STAT3/STAT3 and the IL-37 mRNA levels. IL-37 protein also increased after treatment although its correlation with phosphorylated STAT3 was not significant statistically. This discrepancy might be caused by complicated mechanisms involving multiple processes including the production, processing, transport, and degradation of IL-37 protein. Moreover, considering the unchanged IL37 levels in the plasma of chloroquine-treated monkeys, we speculate that the inductive IL-37 might exert its functions mainly through an intracellular manner.

## 5. Conclusions

In conclusion, we showed that two clinically approved autophagy-modifying drugs, chloroquine and rapamycin, could augment IL-37 expression in LPS-stimulated U937 cells and healthy human PBMCs, probably mediated by LC3 conversion and accumulation. Moreover, we showed that administration of chloroquine alone to rhesus monkeys augmented IL-37 mRNA expression which was inversely correlated with the proportion of proliferating CD4+ T cells. This result implied that IL-37 induction by chloroquine is functional *in vivo* and might be a novel therapeutic mechanism for inflammatory and autoimmune diseases. We also demonstrated the LC3, Erk1/2, and NF-*κ*B/AP-1 pathways as the possible signaling pathways involved in the regulation of IL-37 expression by autophagy-modifying reagents. However, although we identified the statistical correlation between IL-37 mRNA and LC3 II content, the direct evidence supporting the indispensable role of LC3 in IL-37 upregulation is absent. Considering the complicate interactions between LC3 and other autophagic intermediates, there may be tremendous amount of works that is needed to demonstrate that conclusion directly. Moreover, although we supposed that IL-37 mainly exerts its functions through an intracellular manner probably via STAT3 suppression, we need more mechanisms to explain the antiproliferation and antiactivation effects of IL-37.

## Data Availability

The data that support the findings of this study are available from the corresponding author (Guangxing Chen cgx02@hotmail.com), upon reasonable request.

## Supplementary Materials

Supplementary Materials Supplementary Figure 1: immunofluorescence staining of P62/SQSTM1. U937 cells were treated with 400 nM rapamycin, 20 *μ*M chloroquine (CQ), or 1 mM 3-methyladenine (3-MA), respectively, with or without lipopolysaccharide (LPS,200 ng/ml) stimulation for 2 hours. Thereafter, cells were stained with Alexa Fluor 647-conjugated anti-P62/SQSTM1 and observed with a confocal microscope. Neither CQ nor 3-MA treatment induced a significant upregulation of P62/SQSTM1. Supplementary Figure 2: flow cytometry analysis of autophagic U937 cells labeled with Cyto-ID kit. U937 cells were treated with rapamycin, chloroquine, or 3-methyladenine, respectively, with or without lipopolysaccharide stimulation for 2 hours. With flow cytometry analysis, proportion (A) and mean fluorescence density (MFI) (B) of Cyto-ID-labeled autophagic U937 cells were showed in columns. Supplementary Figure 3: sequence logo of matrix profiles with putative binding sites of AP-1. With JASPAR database (http://jaspar.cgb.ki.se), matrix profiles MA0099.2 and MA0476.1 with putative binding sites of AP-1 transcription factors were identified in the IL-37 promoter region. Supplementary Table 1: primer sequences used in this study. Supplementary Table 2: sequences and locations of AP1 putative binding sites within the promotor region of IL-37 analyzed with the JASPAR database.
