# Supplementary material for: Chloroquine and Rapamycin Augment Interleukin-37 Expression via the LC3, ERK, and AP-1 Axis in the Presence of Lipopolysaccharides
Source: J Immunol Res. 2020 Feb 10;2020:6457879. doi: 10.1155/2020/6457879 (PMC7035573; doi:10.1155/2020/6457879)
Supplement: Supplementary Materials — Supplementary Figure 1: immunofluorescence staining of P62/SQSTM1. U937 cells were treated with 400 nM rapamycin, 20 μM chloroquine (CQ), or 1 mM 3-methyladenine (3-MA), respectively, with or without lipopolysaccharide (LPS,200 ng/ml) stimulation for 2 hours. Thereafter, cells were stained with Alexa Fluor 647-conjugated anti-P62/SQSTM1 and observed with a confocal microscope. Neither CQ nor 3-MA treatment induced a significant upregulation of P62/SQSTM1. Supplementary Figure 2: flow cytometry analysis of autophagic U937 cells labeled with Cyto-ID kit. U937 cells were treated with rapamycin, chloroquine, or 3-methyladenine, respectively, with or without lipopolysaccharide stimulation for 2 hours. With flow cytometry analysis, proportion (A) and mean fluorescence density (MFI) (B) of Cyto-ID-labeled autophagic U937 cells were showed in columns. Supplementary Figure 3: sequence logo of matrix profiles with putative binding sites of AP-1. With JASPAR database (http://jaspar.cgb.ki.se), matrix profiles MA0099.2 and MA0476.1 with putative binding sites of AP-1 transcription factors were identified in the IL-37 promoter region. Supplementary Table 1: primer sequences used in this study. Supplementary Table 2: sequences and locations of AP1 putative binding sites within the promotor region of IL-37 analyzed with the JASPAR database. [file 6457879.f1.pdf]

Supplementary Files

Supplementary Figure 1.

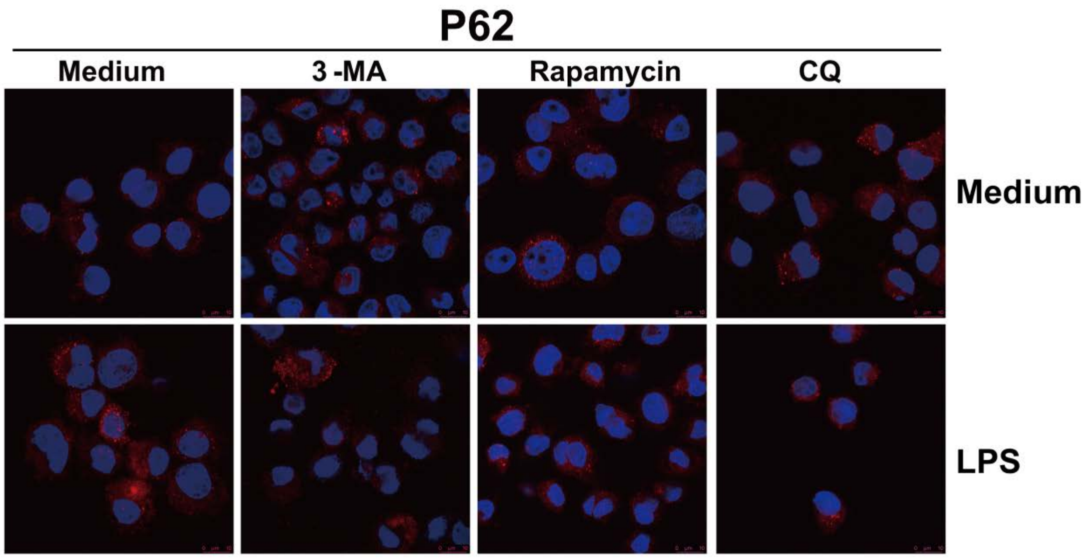

Supplementary Figure 1. Immunofluorescence staining of P62/SQSTM1.

U937 cells were treated with 400 nM rapamycin, 20 μM chloroquine (CQ), or 1 mM 3-methyladenine (3-MA) respectively with or without lipopolysaccharides (LPS,200ng/ml) stimulation for 2 hours. Thereafter, cells were stained with Alexa Fluor 647 conjugated anti- P62/SQSTM1 and observed with a confocal microscope. Neither CQ nor 3-MA treatment induced a significant upregulation of P62/SQSTM1.

Supplementary Figure 2.

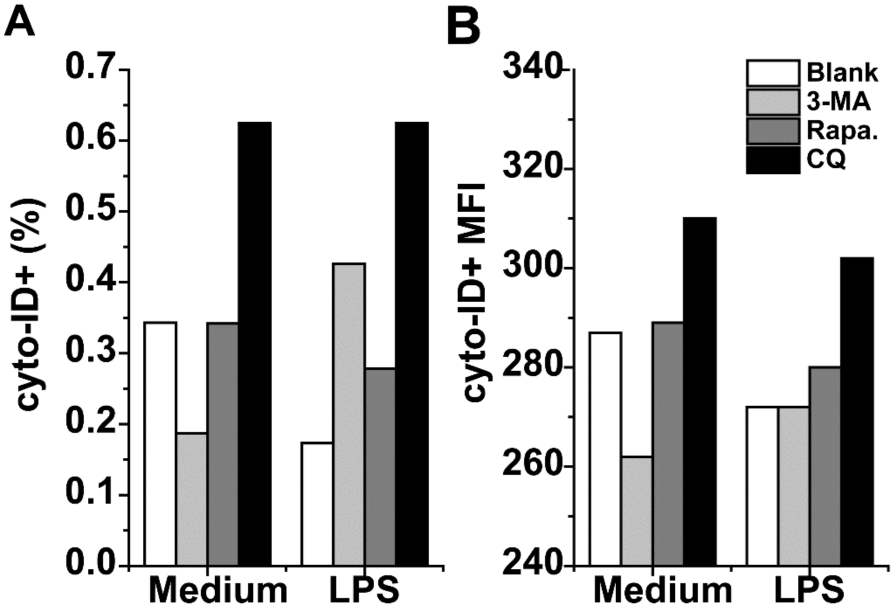

Supplementary Figure 2. Flow cytometry analysis of autophagic U937 cells labeled with Cyto-ID kit.

U937 cells were treated with rapamycin, chloroquine, or 3-methyladenine respectively with or without lipopolysaccharides stimulation for 2 hours. With flow cytometry analysis, proportion (A) and mean fluorescence density (MFI) (B) of Cyto-ID labeled autophagic U937 cells were showed in columns.

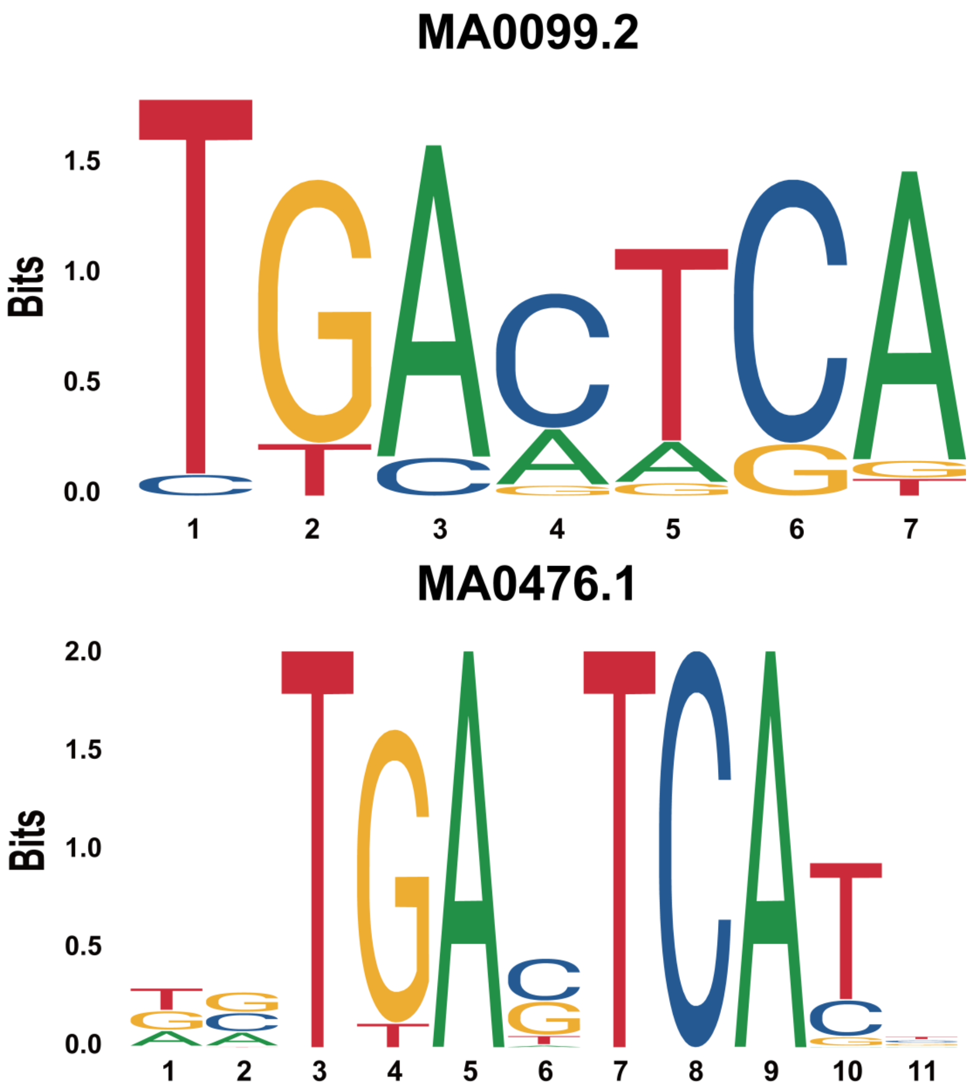

Supplementary Figure 3. Sequence logo of matrix profiles with putative binding sites of AP-1.

With JASPAR database (http://jaspar.cgb.ki.se), matrix profiles MA0099.2 and MA0476.1 with putative binding sites of AP-1 transcription factors were identified in the IL-37 promoter region.

Supplementary Table 1. Primer sequences used in this study.

Primer	Sequence (5′–3′)
Human	
IL-37	F: TTAGAAGACCCGGCTGGAAGCC
	R: AGATCTCTGGGCGTATGTAGT
IL-18	F: ATGGCTGCTGAACCAGTAGAAG
	R: CAGCCATACCTCTAGGCTGGC
IL-6	F: ACTCACCTCTTCAGAACGAATTG
	R: CCATCTTTGGAAGGTTCAGGTTG
TNF-α	F: GGCTCCAGGCGGTGCTTGTTC
	R: AGACGGCGATGCGGCTGATG
IL-1R8	F: TCAGTGGCTCTGAACTGCAC
	R: GTACCAGAGCAGCACGTTGA
IL-18α	F: CACAGACACCAAAAGCTTCATC
	R: CAC AGT CAC TAG GCA CAC TAC
IL-1β	F: ACAGATGAAGTGCTCCTTCCA
	R: GTCGGAGATTCGTAGCTGGAT
GAPDH	F: ACAGTCCATGCCATCACTG
	R: AGTAGAGGCAGGGATGATG
Rhesus macaque	
IL-37	F:TTAGAAGACCCGGCTGGAAGCC
	R:AGATCTCTGGGCGTATGTAGT
GAPDH	F: GTCTGGAAAAACCTGCCAAG
	R: ACCTGGTGCTCAGTGTAGCC

Supplementary Table 2. Sequences and locations of AP1 putative binding sites within the promotor region of IL-37 analyzed with the JASPAR database.

Matrix ID	Name	Score	Relative score	Start	End	Strand	Predicted sequence
MA0099.2	FOS::JUN	10.664	1	221	227	+	TGACTCA
MA0476.1	FOS	10.2377	0.92548	219	229	+	TTTGACTCACC
MA0099.2	FOS::JUN	8.18522	0.909521	257	263	+	TGCCTCA
